# Live imaging of GLUT2 glucose-dependent trafficking and its inhibition in polarized epithelial cysts

**DOI:** 10.1098/rsob.140091

**Published:** 2014-07-23

**Authors:** Merav Cohen, Daniel Kitsberg, Sabina Tsytkin, Maria Shulman, Benjamin Aroeti, Yaakov Nahmias

**Affiliations:** 1 Department of Cell and Developmental Biology, Silberman Institute of Life Sciences, Hebrew University of Jerusalem, Jerusalem, Israel; 2 Alexander Grass Center for Bioengineering, Benin School of Computer Science and Engineering, Hebrew University of Jerusalem, Jerusalem, Israel

**Keywords:** dynamic translocation, polarized renal epithelium, glucose homeostasis, live imaging, phloretin

## Abstract

GLUT2 is a facilitative glucose transporter, expressed in polarized epithelial cells of the liver, intestine, kidney and pancreas, where it plays a critical role in glucose homeostasis. Together with SGLT1/2, it mediates glucose absorption in metabolic epithelial tissues, where it can be translocated apically upon high glucose exposure. To track the subcellular localization and dynamics of GLUT2, we created an mCherry–hGLUT2 fusion protein and expressed it in multicellular kidney cysts, a major site of glucose reabsorption. Live imaging of GLUT2 enabled us to avoid the artefactual localization of GLUT2 in fixed cells and to confirm the apical GLUT2 model. Live cell imaging showed a rapid 15 ± 3 min PKC-dependent basal-to-apical translocation of GLUT2 in response to glucose stimulation and a fourfold slower basolateral translocation under starvation. These results mark the physiological importance of responding quickly to rising glucose levels. Importantly, we show that phloretin, an apple polyphenol, inhibits GLUT2 translocation in both directions, suggesting that it exerts its effect by PKC inhibition. Subcellular localization studies demonstrated that GLUT2 is endocytosed through a caveolae-dependent mechanism, and that it is at least partly recovered in Rab11A-positive recycling endosome. Our work illuminates GLUT2 dynamics, providing a platform for drug development for diabetes and hyperglycaemia.

## 2. Introduction

The facilitative transport of glucose in mammalian cells is mediated by a family of glucose transporters (GLUTs) that show distinct tissue distribution and biochemical properties [1]. GLUT2 is a low-affinity GLUT, expressed predominantly in intestine, liver, kidney and pancreatic β cells, where it mediates critical aspects of glucose homeostasis. GLUT2 plays an important role in the ability of pancreatic β cells to respond to rising glucose levels by secreting insulin [2,3], as well as in postprandial glucose uptake in the intestine and liver [4]. GLUT2 is also the major GLUT in the proximal tubules of the kidney, the main site of glucose reabsorption, where it acts with the Sodium-GLucose Transporter 2 (SGLT2). Loss of GLUT2 in patients suffering from Fanconi–Bickel syndrome results in glycogen accumulation, glucosuria and renal dysfunction [5]. Failure to maintain glucose homeostasis can result in persistent hyperglycaemia, diabetes or cerebral oedema.

Intestinal glucose absorption is mediated by SGLT1, whereas GLUT2 was traditionally considered to enable a basolateral flux [4,6,7]. More recently it was shown that GLUT2 could be apically localized in intestinal cell lines and tissues. The apical translocation of GLUT2 is triggered by membrane depolarization via SGLT1, resulting in a large influx of Ca^2+^, which induces a global cytoskeletal rearrangement, leading to apical localization and activation of PKC βII [8,9]. Insulin was also shown to prevent GLUT2 glucose-dependent apical insertion in mice [10]. Regretfully, renal systems have not been as widely studied as the intestine. Fanconi–Bickel syndrome patients show a mutation in GLUT2 that causes problems in glucose reabsorption and associated renal dysfunction [5]. Apical localization of GLUT2 has been shown in the renal brush border membrane (BBM) of diabetic rats [11], and was correlated with high glucose concentration and PKC βI activation [12].

**Figure 1.**
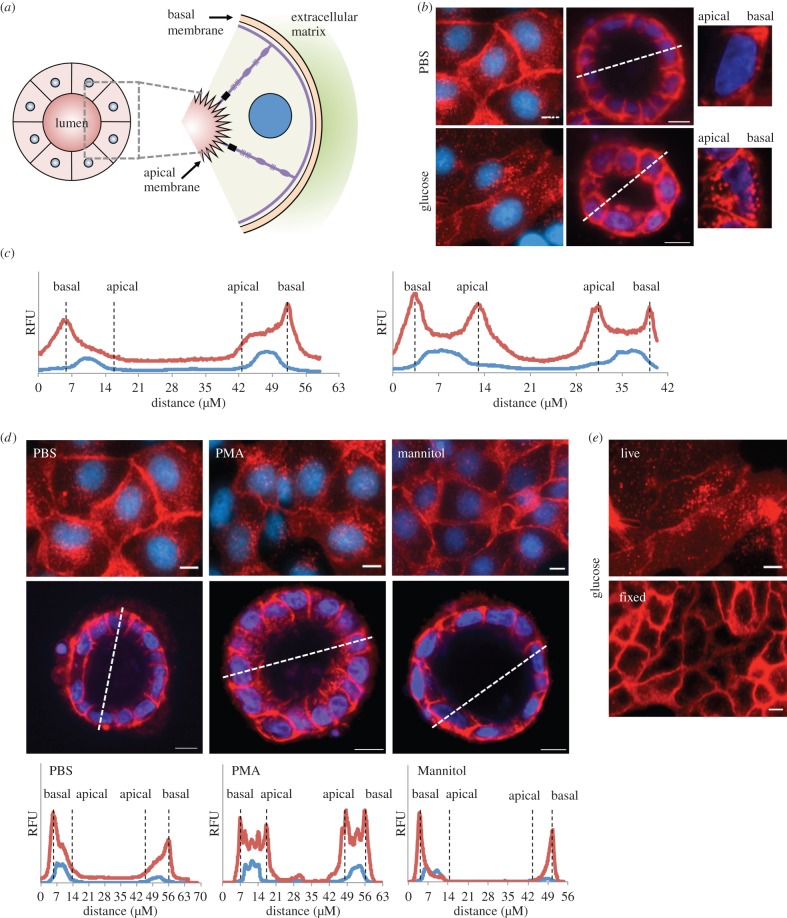
Glucose induces basal to apical re-localization of GLUT2 in MDCK II cell. (*a*) Schematic depiction of multicellular cysts formed in collagen gel. Cysts display an internal apical lumen and an outer basal surface. (*b*) Live imaging of MDCK II cells expressing GLUT2–mCherry fusion protein shows differential localization of GLUT2 in response to 75 mM glucose. Cells in monolayer show perinuclear GLUT2 localization in response to glucose, whereas those in multicellular cysts show redistribution of GLUT2 to the apical membrane. Scale bar, 10 μm. (*c*) Fluorescent profiles of cyst cross-section (dashed lines in (*a*)) show a glucose-induced redistribution of GLUT2 to apical surface. (*d*) Stimulation with 100 nM PMA, a PKC activator, causes perinuclear/apical redistribution of GLUT2, while 75 mM mannitol does not. (*e*) Fixation in 4% paraformaldehyde causes artefactual localization to the membrane even in the presence of 75 mM glucose. Scale bar, 10 μm.

Live cell imaging was previously used to characterize GLUT4 translocation in adipocytes [13], demonstrating insulin-dependent GLUT4 translocation to the plasma membrane. However, tracking GLUT2 localization and dynamics is more difficult due to GLUT2 distinct localization in polarized epithelial architecture. In this work, we used Madin Darby canine kidney (MDCK) type II cells because they were derived from normal kidney and, when cultured in gels of extracellular matrix, can form multicellular structures of polarized epithelium with distinct basolateral and apical surfaces (figure 1*a*). This process was shown to mimic to the physiological development of epithelial kidney tissue *in vivo* [14].

In this work, we established an MDCK II cell line expressing a C-terminal hGLUT2–mCherry fusion protein, which enabled live imaging of glucose-dependent dynamics of GLUT2 in the polarized kidney cells. We show that high glucose exposure results in a PKC-dependent rapid redistribution of GLUT2 from the basolateral to the apical pole of the cells, while the removal of glucose results in a fourfold slower trafficking of GLUT2 to the basal membrane. Interestingly, we show that phloretin, a widely used inhibitor of GLUT2 activity, blocks GLUT2 translocation in both directions. We suggest that the phloretin ability to inhibit PKC activity underlies this effect. Finally, subcellular localization and dynamics show that GLUT2 is endocytosed by a clathrin-independent mechanism and is targeted to Rab11A-positive recycling endosome.

## 3. Material and methods

### 3.1. Reagents

Phenol red-free Dulbecco's modified eagle medium (DMEM), phosphate-buffered saline with Mg^2+^ and Ca^2+^ (PBS), mannitol, phorbol 12-myristate 13-acetate (PMA), phloretin, calphostin C, Filipin and Dynasore were purchased from Sigma Aldrich (St Louis, MO). Fetal Bovine Serum (FBS), l-alanine-l-glutamine, trypsin and sodium pyruvate were ordered from Biological Industries (Beit-Haemek, Israel). Lipofectamine 2000 and G418 antibiotic were purchased from Life Technologies (Carlsbad, CA). EM-grade paraformaldehyde was bought from Polysciences (Warrington, PA), Pitstop2 from ABCAM (Cambridge, UK) and conjugated human holo-transferrin from Jackson Laboratories (Sacramento, CA). Unless otherwise noted, all other reagents were ordered from Sigma Aldrich.

### 3.2. GLUT2–mCherry lines

hGLUT2 coding sequence was amplified from HepG2 hepatoma cells (ATCC) and cloned into XhoI and BamHI restriction sites of pmCherry C1 vector containing G418 resistance cassette (Clonetech, Mountain View, CA). MDCK type II cells (ATCC) were transfected using Lipofectamine 2000 according to manufacturer's protocol and maintained under G418 antibiotic selection. Cells were cultured in DMEM culture medium supplemented with 10% FBS, penicillin/streptomycin, non-essential amino acids and l-alanine-l-glutamine. All cells were cultured under standard conditions (i.e. 37°C in a humidified incubator under 5% CO_2_).

### 3.3. Subcellular compartment reporters

The following constructs were used to transiently transfect MDCK II cells expressing the C-terminal GLUT2–mCherry fusion protein: Rab5A-YFP (kind gift of Mikael Simons, Max Planck Institute of Experimental Medicine, Gottingen, Germany); Rab7A-GFP (kind gift of Benjamin Aroeti, The Hebrew University of Jerusalem, Israel); Rab11A-GFP (kind gift of Jim Goldenring, Vanderbilt University, USA [15]); Furin-CFP (kind gift of Sima Lev, the Weizmann Institute, Israel); Clathrin LC pEGFP (kind gift of Volker Haucke, FMF Berlin, Germany); GFP-C1-TfnR (kind gift of Gary Banker, Center for Research on Occupational and Environmental Toxicology, Oregon Health Sciences University, USA [16]); and Caveolin1-GFP (kind gift of Ari Helenius, ETH Zurich, Switzerland [17]). Plasmid transfection was carried out as described above. Microscopy evaluation of co-localization with GLUT2 mCherry was carried out 12–24 h after reporter transfection.

### 3.4. Madin Darby canine kidney type II spheroid polarization and imaging

MDCK II cells were differentiated into hollow polarized cysts according to the protocol described by Elia & Lippincott-Schwartz [18]. Briefly, collagen gel solution was prepared by mixing 2 mg ml^−1^ rat-tail collagen type-I with 24 mM glutamine, 2.8 mM NaHCO_3_ and 20 mM HEPES buffer, in ice-cold DMEM. The bottom of each 8-well cover-glass slide (Nunc Lab-Tek II) was coated with 45 μl collagen solution by incubating the slide for 30 min at 37°C. GLUT2–mCherry expressing MDCK II cells were trypsinized and added to the collagen solution at a density of 3.7 × 10^4^ cells ml^−1^. Collagen and cell suspension (125 ml) was added to pre-coated wells and allowed to gel for 60 min at 37°C. Culture medium was added to each well and cells were incubated at 37°C, 5% CO_2_ for 4–12 days, with daily media changes, until a central lumen was visible.

### 3.5. GLUT2 translocation

MDCK II cells expressing C-terminal GLUT2–mCherry fusion protein were incubated in PBS with Mg^2+^ and Ca^2+^ (buffer), in the presence or absence of 75 mM glucose, 100 nM PMA or 75 mM mannitol. Cells were counterstained with Hoechst for 1 h before visualization. For the fixation reported in figure 1*e*, 4% paraformaldehyde was added directly to PBS containing 75 mM glucose, incubated for 30 min, briefly washed and visualized.

Time-lapse microscopy of GLUT2 translocation was carried out by replacing PBS buffer with one containing 75 mM glucose or vice versa. Inhibition of GLUT2 translocation was carried out by 30 min pre-incubation with 1 mM phloretin or 50 nM Calphostin C and in the presence of those inhibitors. Cells were counterstained with Hoechst for 1 h before visualization.

### 3.6. GLUT2 endocytosis

To assess clathrin-dependent endocytosis GLUT2–mCherry expressing MDCK II cells were transiently transfected with hTfR-pEGFPC1 construct as described above. Eight hours after transfection, the cells were transferred into DMEM medium supplemented with 0.6% BSA, and cultured overnight. The cells were then incubated for 1 h in PBS buffer to induce externalization of GLUT2 to the plasma membrane, followed by 30 min incubation with 30 μM Pitstop2 or 80 μM Dynasore. Endocytosis of GLUT2 was then induced by exposure to 75 mM glucose in the presence of the inhibitor and 5 μg ml^−1^ AF647-conjugated Transferrin. Internalization of GLUT2–mCherry and AF647-Transferrin was quantified 15 min after induction of endocytosis.

To assess caveolae-dependent endocytosis GLUT2–mCherry expressing MDCK II cells were transiently transfected with CAVEOLIN1-GFP construct as described above. Twenty-four hours after transfection, the cells were incubated for 1 h in PBS buffer to induce externalization of GLUT2 to the plasma membrane, followed by 30 min incubation with 5 μg ml^−1^ Filipin. Endocytosis of GLUT2 was then induced by exposure to 75 mM glucose in the presence of the inhibitor. Cells were imaged 5, 15 and 25 min after induction of endocytosis.

### 3.7. Microscopy

Fluorescence images were taken using a Zeiss LSM 700 imaging system (Carl Zeiss, Germany) equipped with LD Plan Neufluor 20× objective (NA 0.4, WD 7.9 mm). Images were analysed using ZEN 2012 Blue software (Carl Zeiss). Confocal imaging of subcellular co-localization and three-dimensional imaging of spheroids was carried out on the same system in confocal mode using solid-state laser lines 405, 488, 555 and 639 nm. Confocal images were taken with C-Apochromat 40× water immersion objective (NA 1.8, WD 0.28 mm). Analysis was carried out using ZEN 2011 Black software (Carl Zeiss).

### 3.8. Long-term water immersion microscopy

Evaporative loss of water immersion fluid at 37°C was countered by microfluidics. Briefly, a Chemyx Fusion 200 syringe pump was used to perfuse water at a rate of 10 μl min^−1^ through Tygon microtube with a 0.01″ internal diameter. Water formed a droplet at the end of the tubing, and was pulled into the interface between the glass coverslip and the objective by capillary forces. This design permits water immersion microscopy for over 10 h at 37°C.

## 4. Results

### 4.1. Glucose induces basal to apical re-localization of GLUT2 in kidney cysts

GLUT2 cDNA was amplified from human liver cells, and subcloned, in frame, downstream of mCherry coding sequence, driven by a CMV-based promoter. This construct was stably transfected in MDCK type II cells, and the fusion protein was tracked by live cell imaging. Cells cultured in PBS buffer lacking glucose exhibited predominant plasma membrane localization of GLUT2, while exposure to 75 mM glucose resulted in cytoplasmic re-localization (figure 1*b*). To track GLUT2 relevant physiological localization, we cultured GLUT2–mCherry MDCK II cells in collagen gels, allowing them to form multicellular renal cysts with an inner apical surface and an outer basal surface (figure 1*a*) [14]. Cysts cultured in PBS buffer exhibited primarily basal localization of GLUT2, corresponding to two-dimensional plasma membrane localization (figure 1*b*). By contrast, the addition of 75 mM glucose caused re-distribution of GLUT2 to the apical pole of the cells, corresponding to two-dimensional perinuclear localization (figure 1*b*). Quantitative profiles of fluorescence intensities confirmed these basic qualitative observations, as GLUT2 localization in the cysts exhibited a clear redistribution of GLUT2 from basal to apical and basal cytoplasmic regions surrounding the centrally located nucleus (figure 1*c*).

To verify that osmolarity had no effect on GLUT2 localization, we exposed the cells to mannitol. Exposure to 75 mM mannitol did not alter the plasma membrane localization of GLUT2, suggesting that transporter localization was glucose-dependent (figure 1*d*). Previous work suggested that GLUT2 apical insertion in intestinal cells is associated with PKC βII activation [9,19]. Here, we show that exposure to 100 nM PMA, a non-specific PKC activator, promotes similar apical membrane localization of GLUT2 in kidney cells (figure 1*d*).

Interestingly, when comparing the localization of GLUT2 by live imaging to fixed cells, we observed a clear difference. Cells that underwent fixation showed clear plasma membrane localization regardless of glucose concentration (figure 1*e*). Such fixation-induced artefacts were reported in a variety of systems [20] and may have led others to conclude that GLUT2 is localized solely on basolateral surfaces (reviewed in [8]).

### 4.2. Dynamics of GLUT2 translocation

**Figure 2.**
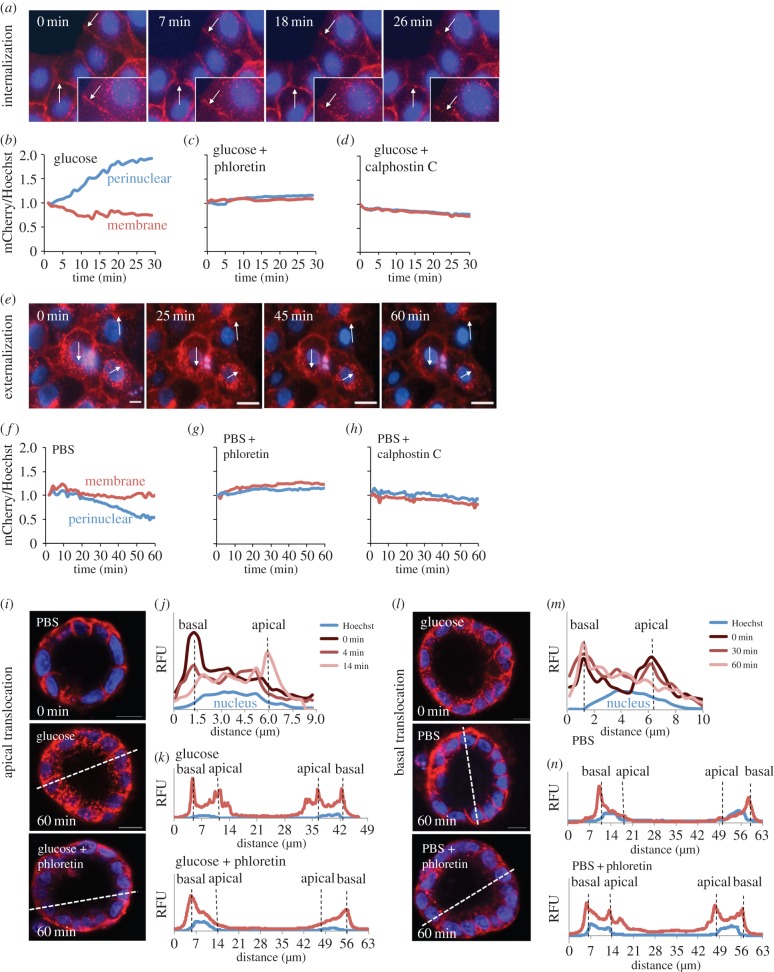
GLUT2 shows a rapid apical translocation in response to PKC-mediated glucose stimulation. (*a*) Live time-lapse microscopy of MDCK II monolayers shows rapid GLUT2 internalization in response to 75 mM glucose. Arrows indicate regions of interest. (*b*) Representative measurement of normalized GLUT2 intensity in perinuclear (blue) or membrane (red) regions shows rapid transporter internalization in response to glucose. (*c*) One millimolar phloretin, an apple polyphenol, blocks GLUT2 redistribution entirely. (*d*) Fifty nanomolar calphostin C, a PKC inhibitor, similarly blocks GLUT2 redistribution. (*e*) Live time-lapse microscopy shows a fourfold slower process of GLUT2 externalization following glucose removal. (*f*) Representative measurement of normalized GLUT2 intensity show slow transporter clearance from the perinuclear region following glucose removal. Both (*g*) 1 mM phloretin and (*h*) 50 nM calphostin C similarly block GLUT2 redistribution. (*i*) Confocal cross-sections of MDCK II cysts show an apical translocation of GLUT2 in response to 75 mM glucose. Apical translocation is abrogated in the presence of phloretin, blocking GLUT2 on the basal surface. (*j*) Time-dependent GLUT2 profile of a single-cell cross-section in a multicellular cyst during glucose-induced apical translocation. Hoechst stain marks the nucleus at the centre of the cell. (*k*) Fluorescent profiles of cyst cross-section (dashed lines) after glucose stimulation, in the presence and absence of 1 mM phloretin. Addition of phloretin blocks the glucose-induced apical translocation. (*l*) Confocal cross-sections of cysts show basal redistribution of GLUT2 in following glucose removal. Basal redistribution is abrogated in the presence of phloretin, blocking GLUT2 on both basal and apical surfaces. (*m*) Time-dependent GLUT2 profile of a single-cell cross-section during glucose absence-induced basal translocation. (*n*) Fluorescent profiles of cyst cross-section (dashed lines) after glucose removal, in the presence and absence of 1 mM phloretin. Addition of phloretin blocks the glucose removal-induced basal translocation. Scale bar, 10 μm.

To determine the dynamics of GLUT2 translocation in MDCK monolayer, we carried out time-lapse microscopy of glucose-induced internalization (figure 2*a*–*d*; electronic supplementary material, movie S1) and glucose removal-induced externalization of the GLUT2 fusion protein (figure 2*e*–*h*; electronic supplementary material, movie S1). GLUT2–mCherry fluorescence was quantified at the plasma membrane (red) and perinuclear (blue) regions of each sequence. Our results showed that glucose-induced internalization was a fast process, peaking at 15.5 ± 2.8 min after glucose stimulations, as seen by the decrease of GLUT2 fluorescence at plasma membrane region coming to a near standstill at this time (figure 2*b*). However, glucose removal resulted in a 3.6-fold slower process of externalization peaking at 55.5 ± 4.0 min after stimulation (figure 2*f*). Interestingly, the majority of GLUT2–mCherry fusion protein was localized to the plasma membrane in two-dimensional cultures, showing minor changes compared with the perinuclear fraction.

To confirm these dynamics in polarized epithelial cysts, we carried out confocal time-lapse imaging in collagen-embedded cysts. PBS buffer was switched to PBS with or without 75 mM glucose as described above. Our results show that glucose-induced translocation to the apical surface was completed in 14 min (figure 2*i*,*j*), whereas glucose removal-induced translocation to the basal surface was fourfold slower, completing in just under 60 min (figure 2*l*,*m*).

### 4.3. Phloretin blocks both basal and apical GLUT2 translocation

Phloretin is a polyphenol found in apple trees' leaves [21], widely used as a glucose transport inhibitor. It is thought that phloretin exerts its effect by binding to GLUT2 and SGLT1 [22,23]. However, other work showed phloretin inhibits PKC [24], suggesting it might directly affect GLUT2 translocation. To test this hypothesis, we performed time-lapse microscopy of glucose-induced internalization (figure 2*c*; electronic supplementary material, movie S2) and glucose removal-induced externalization (figure 2*g*; electronic supplementary material, movie S2) following 30 min of pre-incubation with 1 mM phloretin. We found that both glucose-induced internalization and glucose removal-induced externalization of GLUT2 were inhibited by phloretin (figure 2*c*,*g*).

To confirm phloretin inhibition in polarized kidney cysts, we performed the same experiment on cells embedded in collagen gel. Our results show that 1 mM phloretin causes GLUT2 translocation to the apical membrane to fail in the presence of glucose and we observe GLUT2 localization only the basal (outer) membrane (figure 2*i*,*k*). Similarly, phloretin causes GLUT2 translocation to the basal membrane to fail following glucose removal and we observe GLUT2 on both membranes (figure 2*l*,*n*).

To further investigate the effect of phloretin on GLUT2, and its underlying mechanism, we were able to show that phloretin inhibits GLUT2 internalization and externalization with IC_50_ values of 2.13 ± 0.49 μM and 0.61 ± 0.44 μM, respectively (electronic supplementary material, figure S1*a*). These concentrations are three orders of magnitude lower than the concentration previously used to inhibit glucose uptake by GLUT2 (1 mM; reviewed in [4]). To further assess how phloretin affected GLUT2 mobility, we performed FRAP analysis in the absence or presence of phloretin. Our results show that GLUT2 mobility in the plasma membrane did not change under any condition (electronic supplementary material, figure S1*b*,*c*); however, GLUT2 mobility in the perinuclear region dramatically increased following glucose removal, due to active translocation to the plasma membrane (electronic supplementary material, figure 1*c*). The addition of phloretin reduced GLUT2 mobility at the perinuclear region to its basal level following glucose removal in the presence of glucose (electronic supplementary material, figure S1*c*).

Glucose deprivation and PKC inhibition were previously shown to affect the expression level of GLUTs [25,26]. To rule out phloretin effects on GLUT2 expression, we analysed cells grown in the absence or presence of phloretin for 24 h by FACS and found no significant effect on GLUT2 expression (electronic supplementary material, figure S1*d*).

### 4.4. PKC inhibition blocks GLUT2 translocation

Our results suggest that phloretin blocks GLUT2 translocation, possibly due to PKC inhibition [27]. Interestingly, other PKC inhibitors were similarly shown to inhibit glucose uptake in intestinal cells [28]. We suspected other PKC inhibitors, such as calphostin C, might similarly block GLUT2 translocation. To test this hypothesis, we performed time-lapse microscopy of glucose-induced internalization and glucose removal-induced GLUT2 externalization following 30 min of pre-incubation with 50 nM calphostin C. Again we found that both glucose-induced internalization and glucose removal-induced externalization of GLUT2 were similarly inhibited by treatment with calphostin C (figure 2*d*,*h*). These results suggest that phloretin, calphostin C and other PKC inhibitors might serve as GLUT2 inhibitors due to their ability to block GLUT2 translocation to the appropriate cell membrane.

### 4.5. GLUT2 is internalized by a caveolae-dependent mechanism in kidney cells

**Figure 3.**
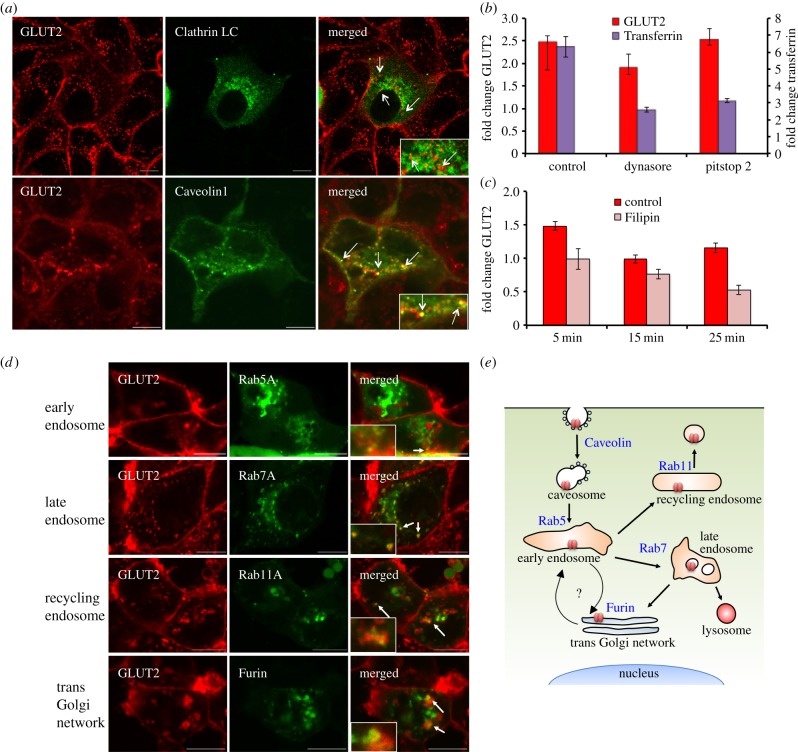
Renal GLUT2 undergoes caveolae-dependent endocytosis and Rab11A-associated endosome recycling. (*a*) Confocal co-localization of GLUT2 (red) and Clathrin LC (green) or Caveolin1 (green) in MDCK II cells. GLUT2 was co-localized with Caveolin1 but not Clathrin LC (white arrows). (*b*) Relative change in GLUT2 and Transferrin endocytosis during glucose-induced internalization, in the presence or absence of inhibitors of clathrin-dependent endocytosis, Dynasore and Pitstop 2. Both inhibitors blocked transferrin endocytosis (**p < 0.01), but do not affect GLUT2 internalization. (*c*) Filipin, a caveolae inhibitor, causes significant inhibition of GLUT2 endocytosis after 15 and 25 min (**p < 0.01). (*d*) Confocal imaging during low-temperature, glucose-mediated internalization shows the co-localization of GLUT2–mCherry fusion protein with Rab5A-YFP (early endosome), Rab7A-GFP (late endosome), Rab11A-GFP (recycling endosome) and Furin-CFP (trans-Golgi network). (*e*) Schematic model for GLUT2 endocytosis and endosome pathway in kidney cells. Renal GLUT2 undergoes caveolae-dependent endocytosis and can be found in all parts of the endosome pathway during internalization, including the recycling endosome. Scale bar, 10 μm; error bars, s.e.m.

Previous work suggested that hepatic GLUT2 is internalized together with the insulin receptor [29] in a clathrin-dependent mechanism [29,30]. To evaluate whether renal GLUT2 is internalized by a similar mechanism, we studied the co-localization of GLUT2–mCherry with GFP-labelled Clathrin light chain (LC) or Caveolin1. We show that GLUT2 co-localized with Caveolin1, but did not co-localize with Clathrin LC (figure 3*a*). We then examined the ability of clathrin-dependent endocytosis inhibitors Dynasore [31] and Pitstop2 [32] to inhibit glucose-induced GLUT2 endocytosis. We found that both inhibitors significantly inhibited the entry of AF647-labelled transferrin, a known cargo of clathrin-coated pits [33]; however, both inhibitors did not affect GLUT2 internalization (figure 3*b*). By contrast, Filipin, an inhibitor of caveolae-dependent endocytosis [34], significantly inhibited glucose-induced GLUT2 internalization (figure 3*c*).

### 4.6. GLUT2 is recycled through the endosome pathway

In Min6 B1 pancreatic cell line, GLUT2 is internalized in response to glucose and undergoes rapid degradation [35,36]. By contrast, hepatic GLUT2 exhibits feeding-mediated internalization to an endosomal pool [37]. To decipher GLUT2 subcellular localization in kidney cells, we studied the co-localization of GLUT2–mCherry with YFP-labelled Rab5A (early endosome marker [38]), GFP-labelled Rab11A (recycling endosome marker [39]), GFP-labelled Rab7A (late endosome marker [40]) or CFP-labelled Furin (trans-Golgi network marker [41]). Endocytosis was induced at 20°C, because slowing the endocytic process allowed the identification of compartments that are briefly occupied by GLUT2. We found that GLUT2 localizes to early endosomes, recycling endosomes, late endosomes and the trans-Golgi network (figure 3*d*). Taken together, our data suggest that glucose stimulation induces caveolae-dependent GLUT2 endocytosis to the endosome pathway from which it is targeted, at least in part, for recycling (figure 3*e*).

## 5. Discussion

In our work, we focused on the mechanism and dynamics of GLUT2 trafficking in the kidney, a major site of glucose reabsorption, and an important pharmaceutical target in diabetes. We established a physiologically relevant system for live imaging of GLUT2 localization and translocation in multicellular cysts of polarized renal epithelial cells (MDCK type II). We show fixation artefact trapping GLUT2 in cellular membrane, highlighting the advantages of live imaging for direct tracking of transporter trafficking. Live imaging also allowed us to quantify GLUT2 dynamics, showing rapid redistribution to the apical membrane following glucose stimulation, compared with a fourfold slower translocation to the basal membrane following glucose removal. These results mark the physiological importance of responding quickly to rising glucose levels. In addition, moving only a fraction of GLUT2 from the basal to the apical surface permits a rapid reabsorption of glucose from the kidney filtrate directly to the blood, bypassing the intracellular compartment.

Pairing our GLUT2–mCherry fusion protein against a library of subcellular markers allowed us to decipher its endocytic pathway in kidney cells. Interestingly, GLUT2 in kidney cells internalize via a caveolae-dependent mechanism, unlike GLUT2 clathrin-mediated entry to liver cells. By contrast, we show that renal GLUT2 enters the endosome system, as in liver cells, and is at least partly recycled in Rab11A-labelled endosomes. This result stands in contrast to pancreatic GLUT2, which is targeted for rapid degradation.

Using live imaging of GLUT4-GFP fusion protein, Fletcher *et al*. [13] were able to unravel much of its translocation mechanism in adipocytes. Live imaging also removes staining artefacts resulting from fixation (as shown here) or from using inappropriate antibodies. One such detected artefact raised some controversy about the localization of GLUT2 to the apical membrane, which resulted from the inability to detect GLUT2 using antibodies raised against the C-terminal of GLUT2 [42], which is masked [43,44] by phosphorylation or interaction with regulatory proteins [45,46].

Phloretin has been known to inhibit transport of chlorine, urea and sugars through membranes by binding to membrane lipid components and altering membrane permeability [47–50]. Phloretin was subsequently found to block sugar uptake in intestinal tissue [51], drawing significant interest to its application in the treatment of diabetes. Phloretin was shown to specifically inhibit GLUT2 and to a lesser extent SGLT1 [22,52]. Here, we show that phloretin inhibits the transport of GLUT2 between the apical and basal membranes in both directions. Our data suggest that phloretin inhibition of GLUT2 might be timing dependent (i.e. taken after a meal, this dietary supplement might in fact increase glucose absorption). Previous work using phloretin to inhibit glucose uptake by GLUT2 in rat intestine was performed using 1 mM phloretin, two orders of magnitude higher than its IC_50_ for inhibition of PKC. In rat intestine, at least, there are two components of apical GLUT2, differing in their trafficking speed and PKC dependence. One millimolar of phloretin was necessary to inhibit absorption by both components at 75 mM glucose, while maintaining specificity [52,53]. Here, we present IC_50_ values for inhibition of GLUT2 translocation by phloretin that suggest that the ability to inhibit PKC activity underlies its inhibition of GLUT2 translocation. Strengthening this hypothesis is the finding that Calphostin C, a potent inhibitor of PKC [54] and glucose uptake [55–57], similarly blocks GLUT2 translocation in both directions. Furthermore, we see no change in GLUT2 mobility in the plasma membrane in the presence of phloretin, but its intracellular mobility was dramatically inhibited, suggesting that the inhibition of GLUT2 translocation is independent of its ability to bind lipid membranes.

In summary, our work elucidates the dynamics of GLUT2 translocation in polarized kidney cells. We show that these dynamics mimic the physiological need to respond fast to changing glucose levels. GLUT2 internalization in kidney cells differs from the liver, possibly due to differences in association with the insulin receptor, which needs to respond faster in liver cells than in kidney to changing insulin and glucose levels. We note that hyperglycaemia causes GLUT2 apical insertion to the BBM of proximal tubule cells [12], and under insulin insensitivity or in lack of insulin, GLUT2 is not internalized [10], leading to increased reabsorption of glucose. MDCK II cells do not express the insulin receptor [58] and therefore mimic proximal tube renal cells in type-I diabetes. We suggest that this system can be used to identify new compounds that inhibit GLUT2 apical localization induced by hyperglycaemia.

## Supplementary Material

Supplementary data and information
