# Supplementary material for: Live imaging of GLUT2 glucose-dependent trafficking and its inhibition in polarized epithelial cysts
Source: Open Biol. 2014 Jul 23;4(7):140091. doi: 10.1098/rsob.140091 (PMC4118605; doi:10.1098/rsob.140091)
Supplement: Supplementary data and information [file rsob140091supp1.pdf]

Dynamics of GLUT2 Glucose-Dependent Trafficking and Its Inhibition

Merav Cohen[1,2], Danny Kitsberg[1,2], Sabina Tsytkin[1], Maria Shulman[1,2], Benjamin Aroeti[1], Yaakov Nahmias[1,2]

1. Department of Cell and Developmental Biology, Silberman Institute of Life Sciences, The Hebrew University of Jerusalem
2. Center for Bioengineering, Benin School of Computer Science and Engineering, The Hebrew University of Jerusalem

SUPPLEMENTARY METHODS

Phloretin inhibition of GLUT2 translocation

Microscopy of GLUT2 translocation was carried out by replacing PBS with $Mg2^+$ and $Ca2^+$ (buffer) to one containing 75 mM glucose or vice versa following 30 minutes pre-incubation with a range of phloretin concentrations and then in the presence of the same concentrations. Cells were counterstained with Hoechst for 1 hour before visualization. Quantification of intracellular GLUT2-mCherry fluorescence and hour after induction of translocation was performed using Image J software (rsbweb.nih.gov). Determination of IC_{50} for phloretin was done using Prism software (GraphPad Software, Inc. La Jolla, California).

FRAP

Imaging was done as described, using the Zeiss LSM 700 imaging system (Carl Zeiss, Germany) with solid state lasers 405 nm 5 mW, 488 nm 10 mW, 555 nm 10 mW and 639 nm 5 mW with an environmental chamber (Okolab, Italy) controlling oxygen, humidity, CO2 and temperature. FRAP was done using a ZEN Dark 2011 using 100% laser intensity. Bleaching was confined to square areas of 15×15 pixels using a C-Apochromat 40x water immersion objective (numerical aperture 1.8, working distance 0.28 mm). Acquisition was done in a 512×512 pixels region. Photobleaching was done in either plasma membrane regions or perinuclear regions, in phenol red free DMEM or PBS. All FRAP analyses were performed on 10-15 cells. Data analyses were as described (1).

FACS analysis

Cells were trypsinized using 0.25% Trypsin EDTA solution (Biological Industries, Israel), centrifuged for 5 minutes at 300xg, and resuspended in PBS (Sigma Aldrich) supplemented with 10% FBS (Biological Industries, Israel). Analysis based on

mCherry fluorescence was done on BD Biosciences FACSAria III using the BD FACSDiva™ Software.

1. Nissim-Rafinia, M., and Meshorer, E. (2011) Photobleaching assays (FRAP & FLIP) to measure chromatin protein dynamics in living embryonic stem cells. *J Vis Exp*

Supplementary Movie 1: GLUT2 Translocation: *Glucose-induced GLUT2 internalization:* arrows indicate breakdown of the solid red lines, corresponding to removal of GLUT2 fusion protein from the plasma membrane. Movie taken for 30 minutes, at 1 frame/minute from time of glucose exposure. *Glucose-removal induced externalization of GLUT2*: arrows indicate increasing space around the nucleus (stained with Hoechst) as GLUT2 fusion protein traffics away from the perinuclear region towards the plasma membrane. Movie taken for 35 minutes, at 1 frame/minute, starting 25 minutes after glucose removal.

Supplementary Movie 2: Phloretin inhibition of GLUT2 translocation: *Glucose-induced GLUT2 internalization with Phloretin*: arrows denote regions of the plasma membrane where GLUT2 translocation inwards is clearly inhibited. Movie taken for 30 minutes, at 1 frame/minute from time of glucose exposure. *Glucose-removal induced GLUT2 externalization with Phloretin*: arrows denote perinuclear regions in which while GLUT2 mobility appears unaffected; its translocation outwards is inhibited. Movie taken for 60 minutes, at 1 frame/minute from time of glucose removal.

SUPLEMENTARY FIGURES

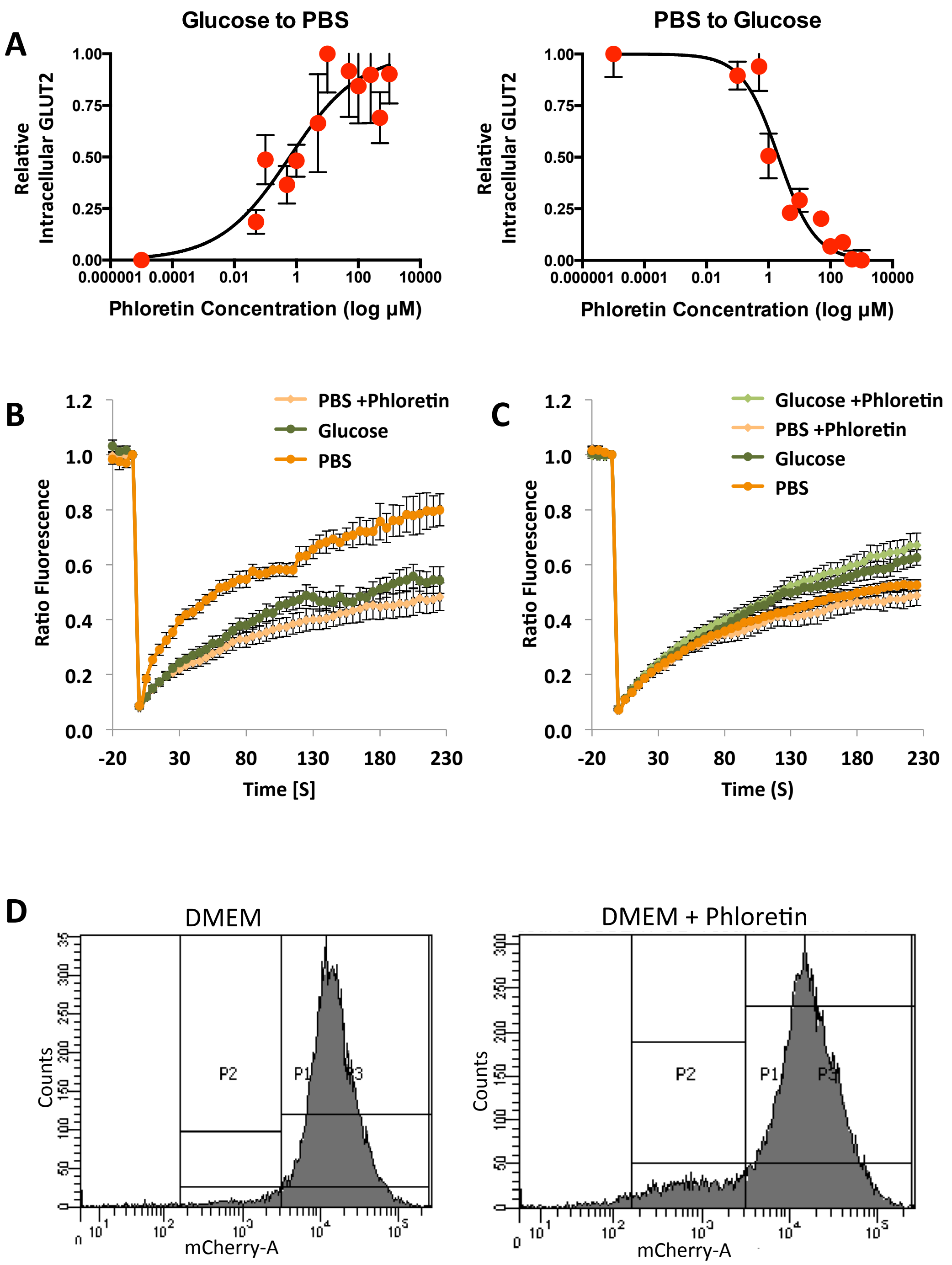

Supplementary Figure1: IC_{50} curves for phloretin inhibition of GLUT2 externalization (left), and internalization (right) **(A).** FRAP analysis of GLUT2 mCherry fusion protein in plasma membrane regions **(B)** and in perinuclear regions **(C)**. FACS analysis of MDCK II cells expressing GLUT2 mCherry fusion protein cultured in the presence or absence of phloretin for 24 hours reveals that phloretin does not significantly change GLUT2 expression (**D**)
